# Neck Collar Assessment for People Living With Motor Neuron Disease: Are Current Outcome Measures Suitable?

**DOI:** 10.2196/43274

**Published:** 2023-03-14

**Authors:** Samuel D J Spears, Yusuf F Abdulle, Dionisios Korovilas, Ryo Torii, Deepak M Kalaskar, Nikhil Sharma

**Affiliations:** 1 Institute of Orthopaedics and Musculoskeletal Science Division of Surgery and Interventional Sciences University College London London United Kingdom; 2 National Hospital for Neurology and Neurosurgery London United Kingdom; 3 Department for Clinical and Movement Neurosciences University College London London United Kingdom; 4 R2 Factory Rolls-Royce London United Kingdom; 5 Department of Mechanical Engineering University College London London United Kingdom

**Keywords:** motor neuron disease, outcome measures, neck collar, bespoke orthoses, 3D scanning

## Abstract

A majority of people living with motor neuron disease (MND) experience weakness of the neck and as a result, experience head drop. This exacerbates problems with everyday activities (eating, talking, breathing, etc). Neck collars are often used to support head drop; however, these are typically designed for prehospitalization settings to manage and brace the cervical region of the spine. As a result, it has been recorded that people living with MND often reject these collars for a variety of reasons but most notably because they are too restricting. The current standardized outcome measures (most notably restricting cervical range of motion) used for neck collars are summarized herein along with whether they are suitable for a bespoke neck collar specifically designed for people living with MND.

## Introduction

Motor neuron disease (MND) is a neurodegenerative disorder that contributes to weakness in the limbs and respiratory and bulbar muscle strength. The disease is irreversible and leads to a fatal outcome typically due to respiratory failure. People living with MND often develop neck weakness. They are unable to keep their head upright, resulting in a clinical syndrome known as a “head drop.” This exacerbates issues with swallowing, breathing, communicating, eating, and drinking. To aid in the management of these symptoms, neck collars are typically used. We will investigate the current issues associated with collars used by people living with MND.

Neck collars have been well established for immobilization [1]. The primary functions include restriction of cervical spine motion, provision of spinal stability, and reduction of pain [2]. Initially, neck orthoses were designed for prehospitalization settings but are now increasingly used to reduce pain and mimic a sense of security for the patient, and to improve postsurgical outcomes [3-5]. Neck collars are used in nontrauma situations; for example, in neurodegenerative diseases such as MND. Despite the redeployment of neck collars in the management of MND, many are rejected by people living with MND due to their restriction on the cervical range of motion (CROM), which can result in unintended outcomes [6,7]. For example, possible muscle atrophy due to complete immobilization of the head accelerates the degradation of muscle tissue [8]. Current neck collars are both an uncomfortable experience and risk-exacerbating issues for people living with MND [7].

The difference in the needs of people living with MND using neck collars and the current aims of standardized collars have resulted in a low uptake of collars by people living with MND [7]. This suggests that outcome measures currently used to assess current neck collars may not be suitable for the design of a new bespoke collar for people living with MND. Therefore, creation of new assessment criteria, specifically for people living with MND, may be necessary. The current use of neck collars will be explored in trauma and MND, as well as outcome measures used to assess its efficacy, and based on the findings, we shall suggest a new way to assess the design of a new bespoke neck collar for people living with MND.

## Trauma Neck Collars and Requirements

**Figure 1:**
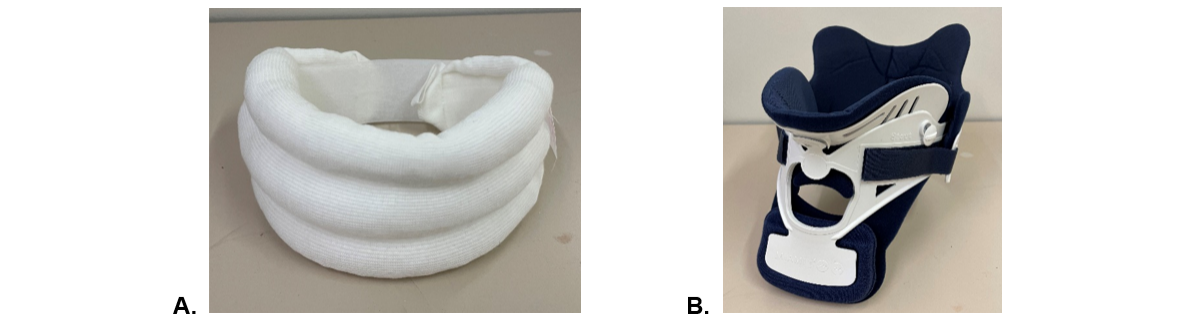
Soft and rigid cervical orthoses: (A) Soft orthosis (Hereford) and (B) rigid orthosis (Miami J).

Neck collars can be classified into 2 types: soft and rigid (Figure 1). Soft collars are designed to be minimally restrictive and to provide the user with a more natural range of motion. These are commonly prescribed to support patients with neck pain and whiplash injuries and are typically made from a thick foam or rubber covered in fabric. Rigid collars are used to restrict range of motion as much as possible, typically applied in severe neck injuries, where there may be a suspected fracture or to stabilize the neck after surgery. These usually consist of a plastic outer shell and a padded inner liner.

Neck collars are typically used to immobilize the spine for suspected spinal injury in prehospital settings as outlined by the National Institute for Health and Care Excellence (NICE) and the Joint Royal Colleges Ambulance Liaison Committee [9,10]. It is recommended by the Joint Royal Colleges Ambulance Liaison Committee and advanced trauma life support that a semirigid collar should be deployed when [9,11] a high-risk factor for cervical spine injury is identified and indicated by the Canadian C-spine rule, and a low-risk factor for cervical spine injury is identified and indicated by the Canadian C-spine rule and the person is unable to actively rotate his/her neck 45° left and right, whereby the collar is only used to stabilize and restrict motion of the cervical and upper thoracic region to prevent further complications from arising.

## MND Neck Collar Requirements

For people living with MND, the NICE guideline for MND (NG42) states that a person experiencing muscle problems ought to be referred to orthotic services as soon as possible and for orthotics to be supplied [12]. The Motor Neurone Disease Association’s (MNDA’s) neck support information sheet outlines that people living with MND can experience different levels of discomfort related to immobility. Thus, it is important that any collar fitted must not create pressure points [13]. A person living with MND may be assessed for a collar by a physiotherapist, occupational therapist, or an orthotist. For many, it will be necessary to try a number of collars on, as problems associated with neck weakness vary due to disease progression, and with current “off the shelf collars,” it is unlikely that one will address all these problems [13].

The MNDA’s *Head supports for motor neurone disease* information sheet [13] states that (1) neck weakness is only part of the problem and is frequently associated with weakness of the shoulder girdle and long back extensor muscles; (2) many people with MND experience swallowing problems as a result of bulbar weakness, and a collar with an anterior area cut away may make swallowing easier; (3) forehead bands give freedom around the chin, mouth, and throat, making it easier to eat, drink, and speak; and (4) practical, easy-to-develop solutions, such as a roll of foam under the chin with a Velcro fastening, can offer some relief in certain circumstances.

For people living with MND requiring wheelchairs and experiencing neck weakness, it is suggested that the preferred position for the person is being tilted with the head, back, and neck supported; therefore, the interaction between the collar and chair must not interfere with one another [13]. It is clear that the requirements for people living with MND experiencing neck weakness differ from prehospitalization applications of neck collars, where MND applications are geared more toward support rather than restriction as needed for trauma.

## Current Neck Collars for People Living With MND

**Figure 2:**
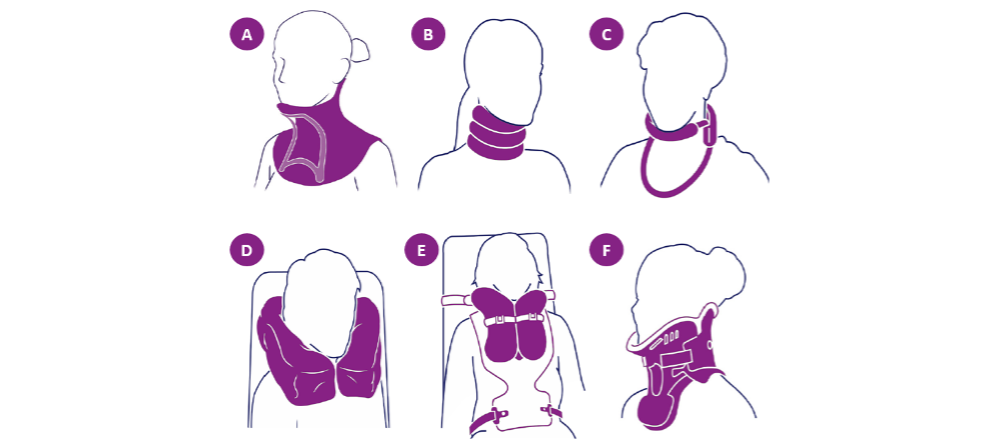
Motor Neurone Disease Association–recommended collars. (A) HeadUp collar, (B) Hereford, (C) Headmaster, (D) Burnett vacuum neck and head supports, (E) Hensinger, and (F) Miami J cervical collar [13].

The current head supports suggested by the MNDA are Soft collar, Wheelchair head supports, HeadUp Collar (Sheffield Support Snood), Hereford, Headmaster, Miami J, and Hensinger [13]. However, the most commonly prescribed collars for people living with MND include Aspen Vista, Philadelphia, Headmaster, HeadUp, Miami J, and Hereford (Figure 2).

**Figure 3:**
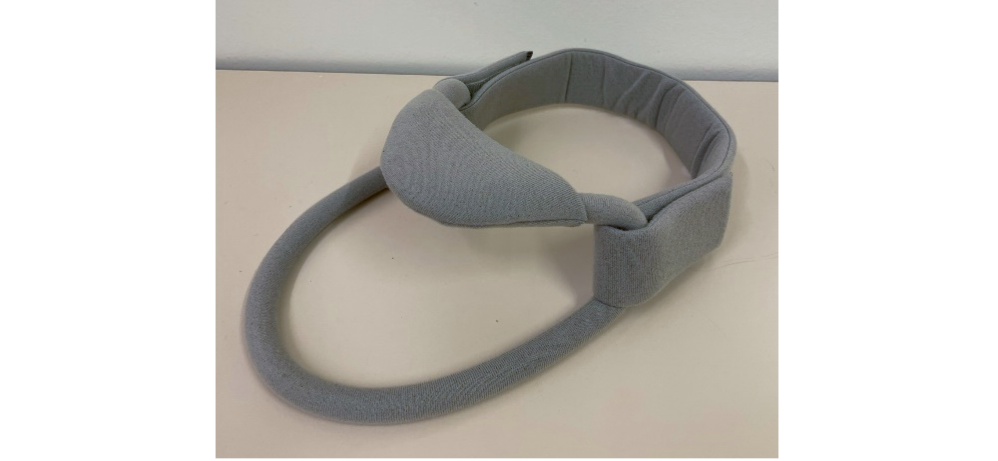
Headmaster collar.

Many of the collars used by people living with MND are rigid with the Hereford and Headmaster being soft and semirigid, respectively. The Headmaster collar (Figure 3) offers a unique design, with a chin rest supported by a semirigid frame and a strap around the neck to hold the orthosis in place. This acts to prevent head drop in the forward plane and does not support the head in other directions, and is often used in conjunction with a headrest for wheelchair users. Baxter et al [14] conducted a survey that investigated MND participants’ experience with existing neck collars, which identified the following themes: “Difficulty fitting,” “lack of physical support,” “overly restrictive,” “uncomfortable,” and “unsuitable.”

## First Orthosis Designed for People Living With MND: HeadUp

The only commercially available collar that has been specifically designed for people living with MND is HeadUp (TalarMade), otherwise known as the Sheffield Support Snood. The HeadUp collar was not assessed by its ability to restrict CROM and was quantitatively measured by assessment of ratio movement coupling (used to normalize the movements of angular velocities in the various planes), to establish control in performing head movements, and angular velocity, to establish whether the collar would compensate (support) the head movements without limiting natural movement velocity [14]. The collar was qualitatively evaluated via interviews or questionnaires against the following criteria:

- Level of support and range of motion
- Appearance
- Fitting the collar
- Breathing, eating, and Swallowing
- Perspiration
- Usage
- Overall satisfaction

While the consensus for the collar was positive, feedback from the interviews conducted by Baxter et al [14] highlighted that eating with the collar on presented issues, with 2 (of 16) participants stating they “can’t possibly eat with it on” and “It makes my swallow harder as it presses on my Adam’s apple,” respectively. Regarding its fit, 2 participants reported that they had issues with the fitting of the collar, 3 reported that they would prefer to use their previous collars, and 1 stated that none of the available collars were satisfactory. Some initial training and practice were required by carers to fit the collar properly. Regarding the questions related to appearance and perspiration, the most frequent answer was neither positive nor negative. It was reported that when the usage of the HeadUp collar was queried among the same cohort of patients, there was no significant difference in the number of hours the collar was used compared to that among participants with a previous collar [14].

This study showed that when designing a collar specifically for people living with MND with neck weakness, the main requirement for the collar was to support head drop without interfering in other daily activities. As such, it may be difficult to design a collar that will be accepted by all, highlighting the importance of direct patient input in the design and outlining requirements for the collar. This can be attributed to differences in disease progression among patients; some report neck weakness only in the sagittal plane while others report weakness in the transverse plane. Also, the degree of neck weakness is due to disease progression, varying from no weakness to severe with the latter, implying that patients are unable to lift their head up whatsoever [14]. Therefore, outcome measures to capture the efficacy of a collar need to allow for a high variance of symptoms, usage, and application.

## Bespoke Collar for People Living With MND

There currently is no fully bespoke neck collars designed for people living with MND, with the closest being the HeadUp, offering custom adjustments to the collar via adjustable supportive strips. A possible alternative is the development of a 3D-printed collar. 3D printing technology allows for the creation of custom-fit, comfortable, and functional orthotic devices. Another major benefit of 3D printing is that it can greatly reduce the lead time and cost associated with traditional methods of manufacturing orthotic devices and at a fraction of the cost of traditional methods [15,16]. The development of a new 3D-printed collar will aim to be fully bespoke, incorporating patient’s anatomy captured via 3D scanning in the design process for each collar. It will also aim to be novel with the inclusion of force sensors to monitor the forces and pressures experienced by the user as this will provide insight into the progression of “head drop” and associated discomfort and whether a new collar is required to be printed to reflect these changes. This novel design will investigate the feasibility of using additive manufacturing and 3D scanning as a viable way to deliver a solution to improve the quality of life for those living with MND and experiencing head drop. 3D scanning can be used to create a detailed model of the patient's anatomy, which can be used to design a custom-fitted orthosis. This technology can also be used to create a digital model of the orthosis, which can be used to test its fit and function before it is manufactured. Finally, by using 3D printing and 3D scanning to design a custom neck collar, outcome measures used to assess the custom collars will be able to capture unique feedback based on each wearer’s “use case,” providing a more accurate efficacy analysis.

## Collar Efficacy Assessment

The efficacy of neck collars has been suggested by several publications with regard to their ability to limit CROM [17]. As neck collars are typically designed for prehospital trauma settings to brace and manage the spine after a spinal injury, surgery, or degenerative changes. These collars are aimed at protecting and providing stability by reducing cervical motion. There are currently a variety of different products available on the market, and a summary of the various studies investigating the effectiveness and outcome measures of these collars is provided in Multimedia Appendix 1 [2,4,5,18-32].

The majority (14/18) of the studies compared sagittal (flexion and extension), transverse (lateral bending), and axial (rotation) planes of motion with and then without an orthosis to see the difference it makes in different planes. Different measurement systems were used to capture the data including goniometric, electromagnetic, optoelectronic, and video fluoroscopy. The subjects recruited for these studies were healthy adults (age range 18-67 years) with no previous history of cervical discomfort or weakness, previous spinal procedures, or pathological condition. All of the studies mentioned, apart from James et al [18] and Schneider et al [19], investigated flexion and extension, lateral bending, and axial rotation movements in a seated position. James et al [18] investigated movements in the supine position and Schneider et al [19] in an upright position.

Only a few studies investigated the impacts of neck orthoses using measures other than CROM. Tescher et al [20] and Plaisier et al [21] highlighted pressure ulcers as a consequence of wearing a collar for extended periods. Worsley et al [22] reported that elevated contact pressures were due to collar design at the device-skin interface with an observed inflammatory response to these increased pressures. Bell et al [23] reported that ill-fitted neck orthoses were unable to provide appropriate restriction of CROM and that orthoses that are too large or too small may cause neck impairment or increase the risk of complications. The lack of variety in collar types and their sizes has meant that there is an increased risk for patients to receive an unsuitable neck collar. Miller et al [5] observed that the efficacy of an orthosis may be reduced if the neck height position is not set correctly and may cause skin-related issues and potential hyperextension.

Collar comfort is also a key factor, as perceived comfort will affect patient compliance to wear and use the collar. Several studies [4,19,22,24] investigated collar comfort in healthy volunteers, with participants asked to rate perceived comfort on a ranking score. A summary of these studies can be found in Multimedia Appendix 2. There currently are no studies comparing collar comfort with people living with MND; therefore, these studies may prove more favorable for collars that immobilize head movement as immobilization may be considered better for aid in pain management.

Other studies suggest that the deployment of neck collars in trauma situations may not provide as much support as previously thought, with some cases actually increasing the risk of medical complications [1]. Extended use of ill-fitting neck collars can lead to increased intracranial and cerebrospinal fluid pressure, causing complications such as pressure ulcers and delirium, and with prolonged collar use, ventilator-associated pneumonia [33]. Therefore, collars should only be deployed for patients with unstable spines, which is difficult to identify in emergency situations [1].

Efficacy in neck collars has traditionally been assessed for trauma and general spine management applications, which emphasizes the need for restriction of head movement, whereas collar needs for people living with MND focus more on support rather than restriction. Applying outcome measures that purely assess CROM restriction are not suitable for collars aimed for people living with MND. However, measuring metrics such as pressure caused by extended periods of use with a collar may prove beneficial in assessing not only the perceived collar comfort by wearers but also good clinical measures that look to prevent pressure ulcers and increased intracranial and cerebrospinal fluid pressure.

## Outcome Measures in Bespoke Ankle Foot Orthoses

The most common orthoses that can be made bespoke are ankle foot orthoses (AFOs) [34]. Foot and ankle problems have meant that older adults display a decreased ability to undertake daily tasks, showing cases of worsening balance, gait, increase in fall risks, and poor health-related quality of life, leading to the development of bespoke AFOs [35]. To better understand and define outcome measures that would be suitable to assess the efficacy of a bespoke neck collar, the relationship between the requirement of bespoke AFOs and the outcome measures used to assess them is investigated.

The Canadian Agency for Drugs and Technologies reported that bespoke AFOs are more effective than prefabricated orthoses when using biomechanical assessments as an objective outcome measure, such as dynamic balance, pressure relief, and load redistribution across plantar regions [36-39].

Heinemann et al [40] performed a survey with orthotists and physiotherapists to establish perspectives of quality-of-life care indicators, for people with bespoke AFOs. In this survey, 60% of participants (461 orthotists and 153 physiotherapists) stated that patient-reported outcome measures were preferred for aspects relating to quality-of-life topics, whereas clinicians were the preferred source for performance-based measures. It was further reported that the current standard assessment instruments are considered “good” by most respondents. The standard assessment measurements in the survey were predominately performance-based. However, it was noted that was there was a conflict between current standard outcome measures and outcome measures preferred by survey respondents, showing a preference for more patient-reported outcome measures to be reported. A separate survey with 257 physiotherapists found that commonly used outcome measures used for bespoke AFOs were a mixture between self-reported outcome measures and performance-based measures: pain assessments, functional tests, and range of motion [41].

A pilot study conducted by Aprile et al [42] investigated the effects of a custom AFO. The outcome measures used were a mixture of performance and patient-reported outcome measures with performance measures consisting of walking performance, stabilometric assessment, and disability; patient-orientated tools consisting of the short-form 36-item questionnaire, the North American Spine Society questionnaire, and the visual analog scale.

Shale [43] reported that patient experiences may be a passable indicator for clinical quality; however, clinical quality indicators may not translate to patient satisfaction. This can be due to complex associations among care assessments, expectations of care, patient knowledge, and objective measures that represent benefit. Wolf et al [44] further agrees that patient satisfaction does not equal patient expectations as expectations vary on a case-by-case basis; therefore, it is important not to alienate other quantitative outcome measures.

Finally, upon understanding how outcome measures are used to assess bespoke AFOs, it is clear that while patient-reported measures aid in understanding quality-of-life metrics for the patient, performance-based measures are still required to provide a clinical and quantitative insight into device performance. Therefore, to assess the design of a bespoke 3D-printed neck collar for people living with MND, there should be both a mixture of patient-reported and performance-based measures to capture both quality-of-life metrics and clinical metrics.

## Discussion

The purpose of this viewpoint article is to describe outcome measures that are currently used to evaluate neck collars and to highlight that current outcome measures for collars are not suitable when applied to the design of a new bespoke collar for people living with MND. The HeadUp collar was the first neck orthosis that was designed specifically for people living with MND to aid in head drop. It focused on performance-based outcome measures to determine efficacy as well as some patent-outcome reported measures.

People living with MND often experience varying levels of progression with the disease; therefore, collar needs differ among patients. It is probable that for people living with MND, the efficacy of the neck orthoses should include patient-reported outcome measures to capture these needs. This highlights that current standardized outcome measures, primarily focusing on restriction for neck orthoses, are not applicable for people living with MND. A combination of new patient-based and performance-based outcome measures for collars designed to suit the individual needs of people living with MND are needed. Patient-focused outcome measures would be suitable in tandem with performance-based outcome measures, which are unable to report patient satisfaction and experience.

For the design of a new bespoke neck collar whose aims are to support and aid in the management of head drop symptoms in people living with MND, the outcome measures used to assess the efficacy should capture individual participants’ responses compared with needs, whereby the collar is assessed on an individual use case compared with a previous collar or no collar (if they have not used one). This would capture participants’ intended use for a collar—with collar use varying case by case—and effectively determine whether or not the collar meets expectations by the user. To do this, it is suggested that patient-reported assessments would be effective in capturing this feedback, by using visual analogue scales and questionnaires along with performance-based measures such as pressure caused by the collar. This would help to not only quantify patient feedback with pressure-associated discomfort but also reduce the risk of pressure-related injuries.

## Conclusions

In conclusion, the current outcome measures used to assess the efficacy of neck collars are not suitable for a bespoke neck collar design. Bespoke orthoses, in general, should take care when outlining their outcome measures as the application and its end user will vary accordingly. Therefore, further work should be conducted to investigate the relationship between the variance experienced by patient expectations and outcome measures used for orthoses.

## Appendix

Multimedia Appendix 1 Studies comparing methods and outcome measures for cervical collars (search conducted on Web of Science using “cervical collars” OR “neck collars” AND “outcome measures”).

Multimedia Appendix 2 Averaged collar comfort scores from [4,18,21,23] (100% being most comfortable). N = No. of studies, n = Sum of No. of participants.

## Abbreviations

AFO: ankle foot orthosis
CROM: cervical range of motion
MND: motor neuron disease
MNDA: Motor Neurone Disease Association
NICE: National Institute for Health and Care Excellence
