# Supplementary material for: Neck Collar Assessment for People Living With Motor Neuron Disease: Are Current Outcome Measures Suitable?
Source: Interact J Med Res. 2023 Mar 14;12:e43274. doi: 10.2196/43274 (PMC10131796; doi:10.2196/43274)
Supplement: Multimedia Appendix 1 [file ijmr_v12i1e43274_app1.pdf]

Appendix 1: Studies comparing methods and outcome measures for cervical collars (search conducted on Web of Science using “cervical collars” OR “neck collars” AND “outcome measures”)

Study	Type of Study	Collars used	Methods	Outcome Measures	References	Study Year
A comfort assessment of existing cervical orthoses	Pilot Study (n=34)	Stro II, Headmaster, Aspen Vista, Philadelphia & Nexus	Survey	Discomfort level associated with orthotic wear & locations of discomfort and participant perceptions about each orthosis	Langley et al. [24]	2018
Investigating the effects of cervical collar design and fit on the biomechanical and biomarker reaction at the skin	Investigating the effects of cervical collar design and fit on the biomechanical and biomarker reaction at the skin (n=15)	Aspen Vista & StifNeck	Interface pressure monitor, humidity & temperature sensor, inflammatory cytokline using Sebutape (IL-1a), Handheld digital inclinometer, 11-point NRS	Interface pressures, inflammatory biomarkers, microclimate, CROM, comfort	Worsely et al. [22]	2018
The Effect of Soft and Rigid Cervical Collars on Head and Neck Immobilization in Healthy Subjects	Semi-experimental study (n=29)	Soft and rigid cervical collar (Teb Sanat)	3D motion analysis	CROM	Barati et al. [25]	2017

Evaluation of clinical efficacy and safety of cervical trauma collars: Differences in immobilization, effect on jugular venous pressure and patient comfort	Randomised crossover design (n=10)	Laerdal Stifneck, Aspen Vista, Miami J Advanced & Philadelphia	Goniometer	Jugular Venous Pressure	Karason et al. [4]	2014
A 3D motion analysis study comparing the effectiveness of cervical spine orthoses at restricting spinal motion through physiological ranges	Randomised crossover design (n=19)	Aspen, Aspen Vista, Philadelphia, Miami-J & Miami-J Advanced	8 Motion Caputure Unit system	CROM	Evans et al. [26]	2013
A comparison of neck movement in the soft cervical collar and rigid cervical brace in healthy subjects	Crossover design (n=50)	Vulkan Medicollar & Combi Collar	Goniometer	CROM	Whitcroft et al. [27]	2011
The effect of rigid cervical collar height on full, active, and functional range of motion during fifteen activities of daily living	Laboratory biomechanical experiment (n=10)	Aspen Vista	electrogoniometer	CROM due to collar heights	Miller et al. [5]	2010

Soft and rigid collars provide similar restriction in cervical range of motion during fifteen activities of daily living	Prospective cohort study (n=10)	Ultra cervical collar & Aspen Vista	electrogoniometer	CROM	Miller et al. [2]	2010
A comparison of three cervical immobilization devices	Prospective laboratory study (n=25)	Xcollar, Ambu Perfit ACE & Jerome NecLoc	Goniometer	CROM	Hostler et al. [28]	2009
Assessing range of motion to evaluate the adverse effects of ill-fitting cervical orthoses	Llaboratory study (n=12)	Miami J	electromagnetic sensor	CROM	Bell et al. [23]	2009
Reduction in head and intervertebral motion provided by 7 contemporary cervical orthoses in 45 individuals	Biomechanical evaluation (n=45)	Philadelphia, Aspen cervical collar, PMT cervical collar, Miami J, Minerva cervicothoracic , Lerman noninvasive halo & Sternal-Occipital-Mandibular-Immobilizer (SOMI)	3D digital tracking sensor & Digital lateral fluroscopy	CROM and comfort	Schneider et al. [19]	2007

Range-of-motion restriction and craniofacial tissue-interface pressure from four cervical collars	Laboratory study (n=48)	Aspen, Philadelphia, Miami J & Miami J with Occian back	Goniometer & XSENSOR X2	CROM and Craniofacial tissue pressure	Tescher et al. [20]	2007
Comparison of cervical spine motion during application among 4 rigid immobilization collars	Randomised controll trial (n=17)	NECLOC, StifNeck, StifNeck Select & Rapid Form Vacuum Immobilizer	Electromagnetic tracking device	AROM	James et al. [18]	2004
Evaluation of efficacy and 3D kinematic characteristics of cervical orthoses	Laboratory biomechanical experiment (n=20)	C-Breeze, XTW, Miami-J & Aspen	3 camera motion analysis ystem	CROM	Zhang et al. [29]	2004
Biomechanical analysis of cervical orthoses in flexion and extension: A comparison of cervical collars and cervical thoracic orthoses	Laboratory biomechanical experiment (n=20)	Miami J, Aspen collar, Aspen 2-post CTO & Aspen 4-post CTO	Optoelectronic motion measurement system and Video Fluroscopy	CROM and Intervetebral ROM	Gavin et al. [30]	2003
Prospective Evaluation of Craniofacial Pressure in Four Different Cervical Orthoses	Laboratory study (n=20)	StifNeck, Philadelphia, Newport & Miami-J	Electropneumatic sensor	Occiput, mandible and chin pressures, comfort	Plaisier et al. [21]	1994

Comparison of two new immobilization collars	Laboratory study (n=15)	Nec-Loc Extrication Collar, Philadelphia Collar, Philadelphia Red EM Collar with Immobilizer & Vacuum Splint Cervical Collar	Head Goniometer and handheld goniometer	CROM	Rosen et al. [31]	1992
Restriction of neck flexion using soft cervical collars: a preliminary study	Randomised controll trial (n=40)	Airway soft cervical collars & handmade cervical roughs	Spinal Rangiometer	CROM	Aker et al. [32]	1991
