# Supplementary figures and images for: Neck Collar Assessment for People Living With Motor Neuron Disease: Are Current Outcome Measures Suitable?
Source: Interact J Med Res. 2023 Mar 14;12:e43274. doi: 10.2196/43274 (PMC10131796; doi:10.2196/43274)

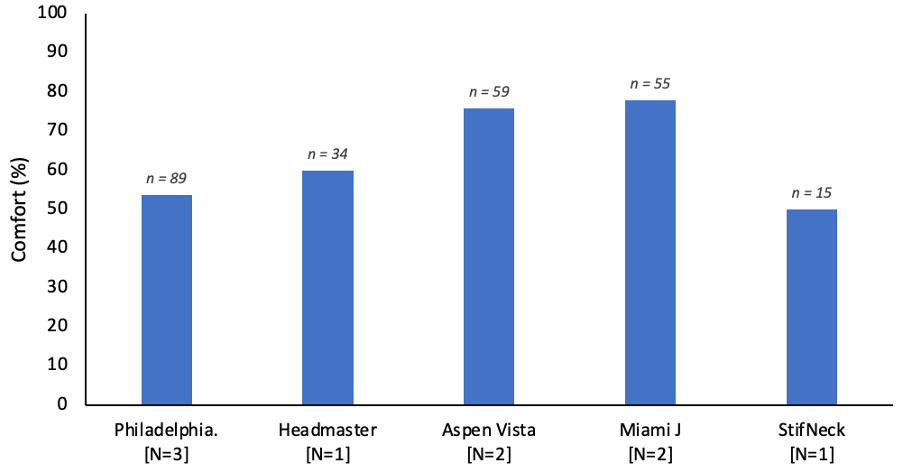

Supplement: Multimedia Appendix 2 [file ijmr_v12i1e43274_app2.png]
